# Estrogen receptor α promotes Cav1.2 ubiquitination and degradation in neuronal cells and in APP/PS1 mice

**DOI:** 10.1111/acel.12961

**Published:** 2019-04-22

**Authors:** Yu‐Jie Lai, Bing‐Lin Zhu, Fei Sun, Dong Luo, Yuan‐Lin Ma, Bio Luo, Jing Tang, Ming‐Jian Xiong, Lu Liu, Yan Long, Xiao‐Tong Hu, Ling He, Xiao‐Juan Deng, John H. Zhang, Jian Yang, Zhen Yan, Guo‐Jun Chen

**Affiliations:** ^1^ Department of Neurology, Chongqing Key Laboratory of Neurology the First Affiliated Hospital of Chongqing Medical University Chongqing China; ^2^ Department of Neurology the Third Affiliated Hospital of Chongqing Medical University Chongqing China; ^3^ Department of Physiology Wayne State University School of Medicine Detroit Michigan; ^4^ Division of Physiology, School of Medicine Loma Linda University Loma Linda California; ^5^ Department of Biological Sciences Columbia University New York City New York; ^6^ Department of Physiology and Biophysics State University of New York at Buffalo Buffalo New York

**Keywords:** Alzheimer’s disease, Cav1.2, Estrogen receptor α, K29, Mdm2, ubiquitination

## Abstract

Cav1.2 is the pore‐forming subunit of L‐type voltage‐gated calcium channel (LTCC) that plays an important role in calcium overload and cell death in Alzheimer's disease. LTCC activity can be regulated by estrogen, a sex steroid hormone that is neuroprotective. Here, we investigated the potential mechanisms in estrogen‐mediated regulation of Cav1.2 protein. We found that in cultured primary neurons, 17β‐estradiol (E2) reduced Cav1.2 protein through estrogen receptor α (ERα). This effect was offset by a proteasomal inhibitor MG132, indicating that ubiquitin–proteasome system was involved. Consistently, the ubiquitin (UB) mutant at lysine 29 (K29R) or the K29‐deubiquitinating enzyme TRAF‐binding protein domain (TRABID) attenuated the effect of ERα on Cav1.2. We further identified that the E3 ligase Mdm2 (double minute 2 protein) and the PEST sequence in Cav1.2 protein played a role, as Mdm2 overexpression and the membrane‐permeable PEST peptides prevented ERα‐mediated Cav1.2 reduction, and Mdm2 overexpression led to the reduced Cav1.2 protein and the increased colocalization of Cav1.2 with ubiquitin in cortical neurons in vivo. In ovariectomized (OVX) APP/PS1 mice, administration of ERα agonist PPT reduced cerebral Cav1.2 protein, increased Cav1.2 ubiquitination, and improved cognitive performances. Taken together, ERα‐induced Cav1.2 degradation involved K29‐linked UB chains and the E3 ligase Mdm2, which might play a role in cognitive improvement in OVX APP/PS1 mice.

## 1 INTRODUCTION

Cav1.2 is the pore‐forming subunit of L‐type voltage‐gated calcium channels (LTCC) and accounts for approximately 70% of LTCC in the brain (Zamponi, Striessnig, Koschak, & Dolphin, 2015). Cav1.2 is mainly located at extra‐ and postsynaptic sites and plays a critical role in intracellular calcium transients (Tippens et al., 2008). During aging, LTCC activity is increased, and calcium influx via LTCC may be the preferred source for calcium‐induced calcium release (CICR), which plays a necessary role in aging‐related biomarkers (Thibault, Gant, & Landfield, 2007). Evidence has suggested that dysfunction of LTCC and calcium overload contribute to cell death and the pathophysiology of Alzheimer's disease (AD) (Bezprozvanny & Mattson, 2008). Accordingly, LTCC inhibitors have exhibited therapeutic potential in AD (Nimmrich & Eckert, 2013).

Estrogens are neuroprotective in brain aging and AD (Engler‐Chiurazzi, Brown, Povroznik, & Simpkins, 2016). Estrogen deficiency promotes the generation of the toxic amyloid β protein (Aβ) (Yue et al., 2005), supporting the role of estrogens in AD‐like pathologies. Among the multiple mechanisms underlying estrogen action in the brain (Nilsson et al., 2001), the involvement of LTCC‐mediated calcium dynamics has been documented (Vega‐Vela et al., 2016). Brief exposure (90 s) of 17β‐estradiol (E2) induces rapid increase in LTCC currents, which is mediated by direct interaction of E2 with LTCC subunit (Sarkar et al., 2008). Interestingly, estrogen also induces rapid release of calcium from intracellular store within minutes in isolated neurons, possibly by mechanism of CICR (Beyer & Raab, 1998; Thibault et al., 2007). On the other hand, long‐term estrogens can inhibit LTCC in neuronal cells. For instance, E2 (25 hr) inhibits the high‐voltage‐activated calcium channel currents, which are associated with LTCC (Kumar & Foster, 2002). In glutamate‐ or high potassium‐primed neurons, E2 (5 min ~ 25 hr) reduces calcium entry and cell death through LTCC (Kurata, Takebayashi, Kagaya, Morinobu, & Yamawaki, 2001; Sribnick, Re, Ray, Woodward, & Banik, 2009). Chronic E2 replacement prevents the age‐related increase of LTCC currents in the hippocampus of ovariectomized (OVX) rats (Brewer et al., 2009). Therefore, identifying the molecular mechanisms underlying estrogen regulation of Cav1.2 may aid to the understanding and treatment of AD.

In the current study, we found that E2 reduced Cav1.2 protein through estrogen receptor α (ERα). This effect involved lysine 29‐linked ubiquitin chains and the E3 ligase Mdm2 (double minute 2 protein). In OVX APP/PS1 mice, systematic administration of E2 and ERα agonist PPT (propylpyrazoletriol) led to the reduced Cav1.2 protein and the enhanced Cav1.2 ubiquitination in the brain, which were accompanied by the improved cognitive functions.

## 2 RESULTS

### 2.1 Estrogen reduced Cav1.2 protein through ERα in primary neurons

**Figure 1:**
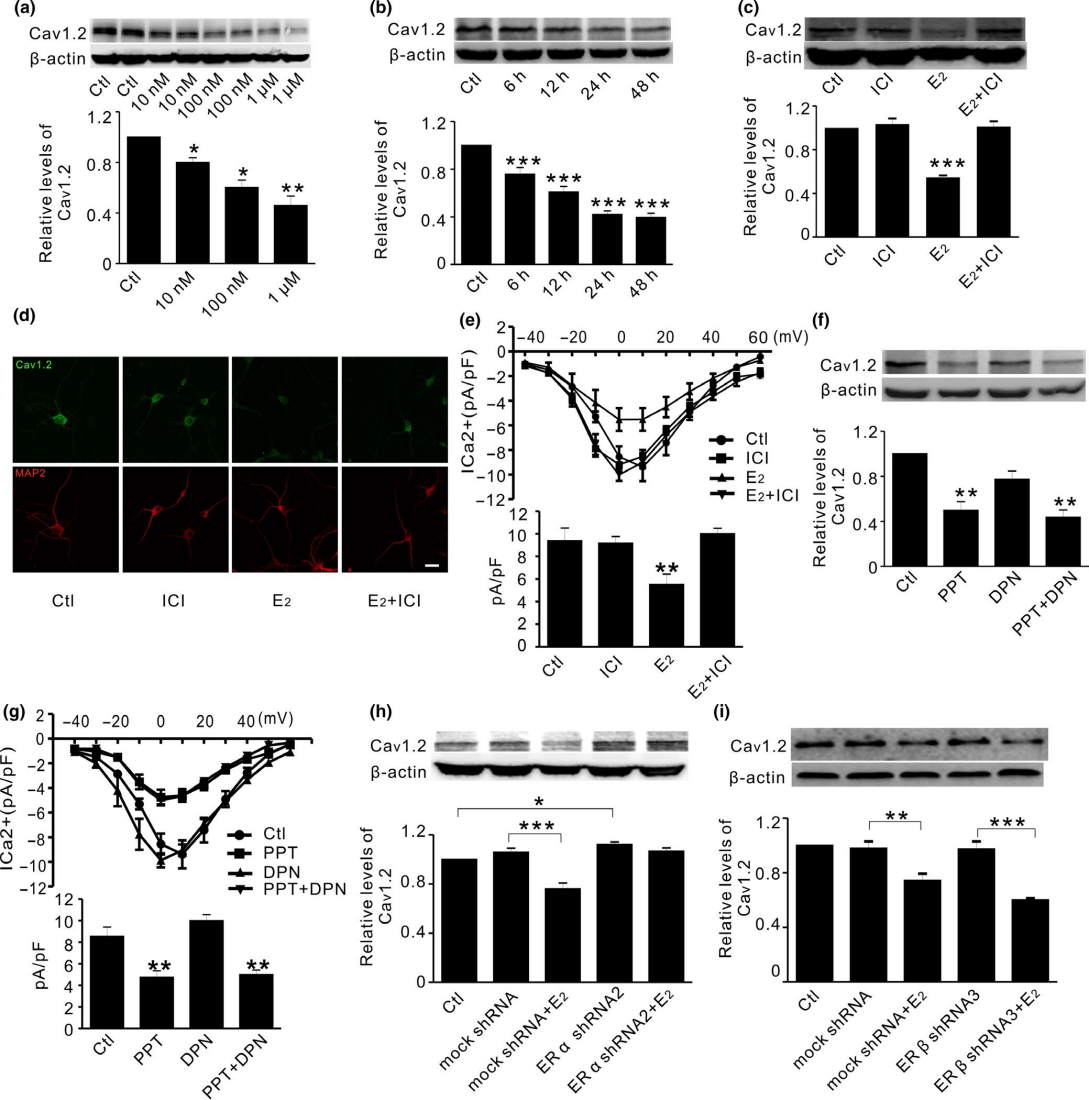
Estrogen decreases Cav1.2 protein through ERα in primary cortical neurons. (a) Representative Western blots (top) and quantification (bottom) of Cav1.2 in primary cortical neurons in the absence (Ctl) and presence of estrogen at 10, 100 nM, and 1 μM, respectively; *n* = 4. (b) Representative Western blots (top) and quantification (bottom) of Cav1.2 in cortical neurons incubated with estrogen (100 nM) at different times; *n* = 3*.* (c) Representative Western blots (top) and quantification (bottom) of Cav1.2 in cortical neurons treated with DMSO (Ctl), the ER antagonist ICI182780 (1 μM, ICI), estrogen (100 nM, E2), and E2 with ICI, respectively *n* = 4*.* (d) Representative immunofluorescent images of Cav1.2 (green) relative to neuronal marker MAP2 (red), in cortical neurons treated with DMSO (Ctl), ICI, E2, and E2 with ICI for 24 hr (*n* = 3). Scale bar, 10 μm*.* (e) I‐V plots (top) and quantifications (bottom) of calcium mediated current (ICa^2+^) density (pA/pF) in primary cortical neurons treated with DMSO (Ctl, *n* = 15), ICI (*n* = 9), E2 (*n* = 9), and E2 with ICI (*n* = 11) for 24 hr. (f) Representative Western blots (top) and quantification (bottom) of Cav1.2 in primary cortical neurons treated with ERα agonist PPT (10 nM), ERβ agonist DPN (10 nM), and PPT with DPN for 24 hr; *n* = 6. (g) I‐V plots (top) and quantifications (bottom) of ICa^2+^ density (pA/pF) in cortical neurons treated with DMSO (Ctl, *n* = 15), PPT (*n* = 9), DPN (*n* = 9), and PPT with DPN (*n* = 11). (h) Representative Western blots (top) and quantification (bottom) of Cav1.2 in cortical neurons in the absence (Ctl) and presence of mock shRNA, mock shRNA with estrogen (E2, 100 nM), ERα shRNA2, and ERα shRNA2 with E2, respectively (*n* = 4). (i) Representative Western blots (top) and quantification (bottom) of Cav1.2 in cortical neurons in the absence (Ctl) and presence of mock shRNA, mock shRNA with estrogen (E2, 100 nM), ERβ shRNA3, and ERβ shRNA3 with E2, respectively (*n* = 3). *p＜0.05,**p＜0.01,***p＜0.001 (ANOVA)

As shown in Figure 1a and b, 17β‐estradiol (E2) significantly reduced Cav1.2 expression at concentrations ranging from 10 nM to 1 μM. Cav1.2 protein level was decreased at 6 hr and remained to be decreased for up to 48 hr in primary neurons incubated with E2. This effect seemed to be dependent on estrogen receptors (ERs), as a nonselective ER antagonist fulvestrant (ICI182780, ICI, 1 μM) diminished the effect of E2 on Cav1.2 (Figure 1c). Similar results were found by immunofluorescent studies (Figure 1d). To further assess whether E2 may affect Cav1.2 function, the voltage‐gated calcium currents (I_Ca_^2+^density) were measured using whole‐cell patch recordings. As shown in Figure 1e, the peak I_Ca_^2+^density (pA/pF) was significantly decreased by E2 treatment (24 hr), whereas ICI attenuated this effect. These results indicated that E2 reduced Cav1.2 protein in cultured primary cortical neurons, which was mediated by ERs.

To identify which ER subtype(s) might mediate E2 effect on Cav1.2, we tested the effect of the selective ERα agonist (PPT, 10 nM) or ERβ agonist (diarylprepionitrile, DPN, 10 nM) on Cav1.2 incubated in cortical neurons for 24 hr. As shown in Figure 1f, PPT but not DPN alone significantly reduced Cav1.2 expression. Combined incubation with PPT and DPN had no additive effect, compared with PPT alone. Consistently, PPT but not DPN significantly reduced I_Ca_^2+^ density (Figure 1g). The involvement of ER subtype was further assessed using lentivirus bearing ERα or ERβ shRNA. Primary screening with three different sequences targeting ERα or ERβ mRNA showed that ERα shRNA2 and ERβ shRNA3 were most effective in reducing ERα or ERβ protein, respectively (Figure S1). As expected, ERα shRNA2 significantly increased Cav1.2, which attenuated E2‐induced reduction of Cav1.2 (Figure 1h). In contrast, ERβ shRNA3 did not alter the basal Cav1.2 and failed to further attenuate Cav1.2 reduction by E2 (Figure 1i). These results indicated that ERα mediated E2 reduction of Cav1.2.

### 2.2 ERα regulation of Cav1.2 involved K29‐linked ubiquitin chains

Cav1.2 protein can be regulated at multiple levels (Dai, Hall, & Hell, 2009). It was unlikely that ERα may affect Cav1.2 at transcriptional level, as Cav1.2 mRNA was not changed (Figure S2A and B). Moreover, ERα agonist PPT also failed to alter Cav1.2 phosphorylation. And the level of the calcineurin (PP2B, CN), the phosphatase involved in Cav1.2 dephosphorylation (Oliveria, Dell'Acqua, & Sather, 2007), was not altered (Figure S2C and D). Although PPT enhanced CN association with Cav1.2, CN inhibitor FK506 did not prevent PPT effect on Cav1.2 in primary neurons (Figure S2E and F). Thus, it was also unlikely that the phosphorylation‐related mechanisms are involved in ERα‐mediated Cav1.2 reduction.

**Figure 2:**
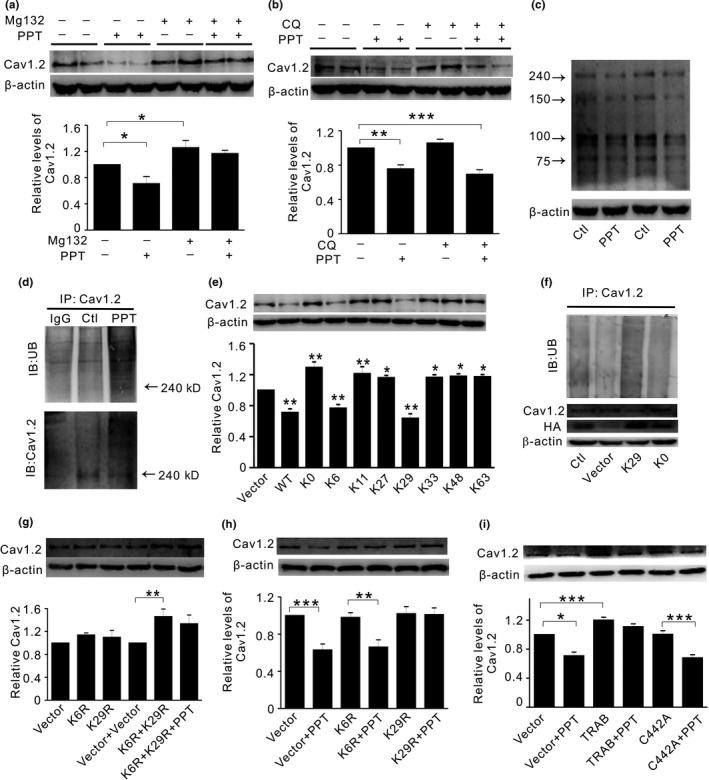
K29‐linked UB chains are involved in ERα‐induced Cav1.2 ubiquitination and degradation. (a) Representative Western blots (top) and quantification (bottom) of Cav1.2 in primary neurons treated with PPT in the absence or presence of proteasomal inhibitor Mg132 (10 μM for 24 hr, *n* = 7). (b) Representative Western blots (top) and quantification (bottom) of Cav1.2 in cortical neurons treated with PPT in the absence or presence of lysosomal inhibitor chloroquine (CQ, 50 μM for 24 hr, *n* = 10). (c) Representative Western blots of the predicted molecular masses of full‐length and three fragments of Cav1.2 protein naturally occurred in primary cortical neurons, which shows that PPT decreases all fragments of Cav1.2 (*n* = 4). (d) Western blots of ubiquitin (UB, top) and Cav1.2 (bottom) in primary cortical neurons immunoprecipitated by Cav1.2 antibody under control conditions or after PPT treatment. (e) Representative Western blots (top) and quantification (bottom) of Cav1.2 in HT22 cells transiently expressing vector, or ubiquitin constructs WT, K0, K6, K11, K29, K33, K48, and K63, respectively (*n* = 4). In WT and K0, all 7 lysine (K) residues were included or mutated to arginine (R), respectively. In K6‐K63, only the numbered K residue was present, while the rest 6Ks were mutated to Rs. Overexpression of the ubiquitin mutant K6 or K29 significantly reduces Cav1.2 expression compared with control. (f) Top: Western blots of ubiquitin in HT22 cells without treatment (Ctl), or transiently expressing vector, K29 or K0 constructs, after cell extracts were immunoprecipitated by Cav1.2 antibody. Bottom: corresponding Western blots of Cav1.2 and HA tag under these conditions. (g) Representative Western blots (top) and quantification (bottom) of Cav1.2 in HT22 cells transiently expressing vector, mutant ubiquitin construct K6R and K29R alone or in combination, in the absence and presence of PPT, respectively (*n* = 3). (h) Representative Western blots (top) and quantification (bottom) of Cav1.2 in HT22 cells transiently expressing vector, mutant ubiquitin construct K6R or K29R, in the absence and presence of PPT, respectively (*n* = 5). (i) Representative Western blots (top) and quantification (bottom) of Cav1.2 in HT22 cells transiently expressing vector, wild‐type TRABID or mutated TRABID (C442A) construct, in the absence and presence of PPT, respectively (*n* = 4).*p＜0.05,**p＜0.01,***p＜0.001, ANOVA. TRABID: TRAF‐binding protein domain

It is reported that ubiquitin–proteasome system (UPS) controls Cav1.2 degradation (Altier et al., 2011). Thus, we compared the effect of the proteasomal inhibitor MG132 (10 μM for 24 hr) and the lysosomal inhibitor chloroquine (CQ, 50 μM for 24 hr) in primary neurons. As shown in Figure 2a and b, MG132 significantly increased Cav1.2 under basal condition and diminished PPT‐induced reduction of Cav1.2, whereas CQ was without effect. Accordingly, the naturally occurred Cav1.2 fragments in primary neurons (Michailidis et al., 2014) were all decreased by PPT (Figure 2c). Further co‐immunoprecipitation experiments showed that Cav1.2 ubiquitination was significantly increased by PPT (Figure 2d). These results suggested that UPS played a role in ERα regulation of Cav1.2.

Ubiquitin (UB) protein contains seven lysine (K) residues at positions 6, 11, 27, 29, 33, 48, and 63, respectively. In addition, poly‐UB chain assembly can occur at any of these lysine residues (Pickart & Fushman, 2004). To further identify which K(s) may be involved in ERα regulation of Cav1.2, we assessed Cav1.2 protein expression in HT22 (hippocampal neuron) cell line transiently transfected with UB mutants WT, K0, K6, K11, K29, K33, K48, and K63, respectively. In WT (wild‐type) and K0, all 7Ks were included or mutated to arginine (R), respectively. In K6‐K63, only the numbered K residue was present, while the rest 6Ks were mutated to Rs. As shown in Figure 2e, a significant reduction of Cav1.2 was seen in WT. In contrast, in K0‐transfected cells, Cav1.2 was significantly increased compared to control. Similar increases of Cav1.2 were shown in K11, K27, K33, K48, and K63 compared with control. Only K6‐ and K29‐expressing cells exhibited significantly reduced Cav1.2 relative to control (Figure 2e), suggesting that K6/K29 was involved in Cav1.2 degradation. The decreased Cav1.2 protein might be associated ubiquitination, as the enhanced Cav1.2 ubiquitination coincided with the reduced Cav1.2 protein in K29‐overexpressing cells, relative to K0 or vector control (Figure 2f). Interestingly, mutation of K6 (K6R) or K29 (K29R) alone did not increase Cav1.2 protein; unless both K6R and K29R were introduced (Figure 2g), suggesting that efficient Cav1.2 degradation required poly‐UB chains under basal condition. To test whether K6/K29 might be involved in ERα regulation of Cav1.2, we assessed Cav1.2 protein in HT22 cells overexpressing K6R or K29R. As shown in Figure 2h, overexpression of K29R alone, but not K6R alone, was sufficient to attenuate PPT‐induced Cav1.2 reduction. These results indicated while both K6 and K29 were required for efficient Cav1.2 degradation under basal condition, K29R alone seemed to be sufficient to attenuate PPT‐induced Cav1.2 degradation.

K29‐linked UB chains can be specifically regulated by the deubiquitinating enzyme TRABID (TRAF‐binding protein domain) (Kristariyanto et al., 2015) and TRABID mutant C443A that binds to but does not hydrolyze K29‐linked UB chains (Kristariyanto et al., 2015). As shown in Figure 2i, in TRABID‐transfected cells, PPT‐induced reduction of Cav1.2 was diminished. In contrast, C443A overexpression did not significantly affect PPT‐induced reduction of Cav1.2. Together, these results indicated that K29‐linked UB chains were critical in mediating ERα‐induced ubiquitination and degradation of Cav1.2 in neuronal cells.

### 2.3 The E3 ligases Mdm2 and the pest sequence in Cav1.2 were Involved in ERα regulation of Cav1.2

**Figure 3:**
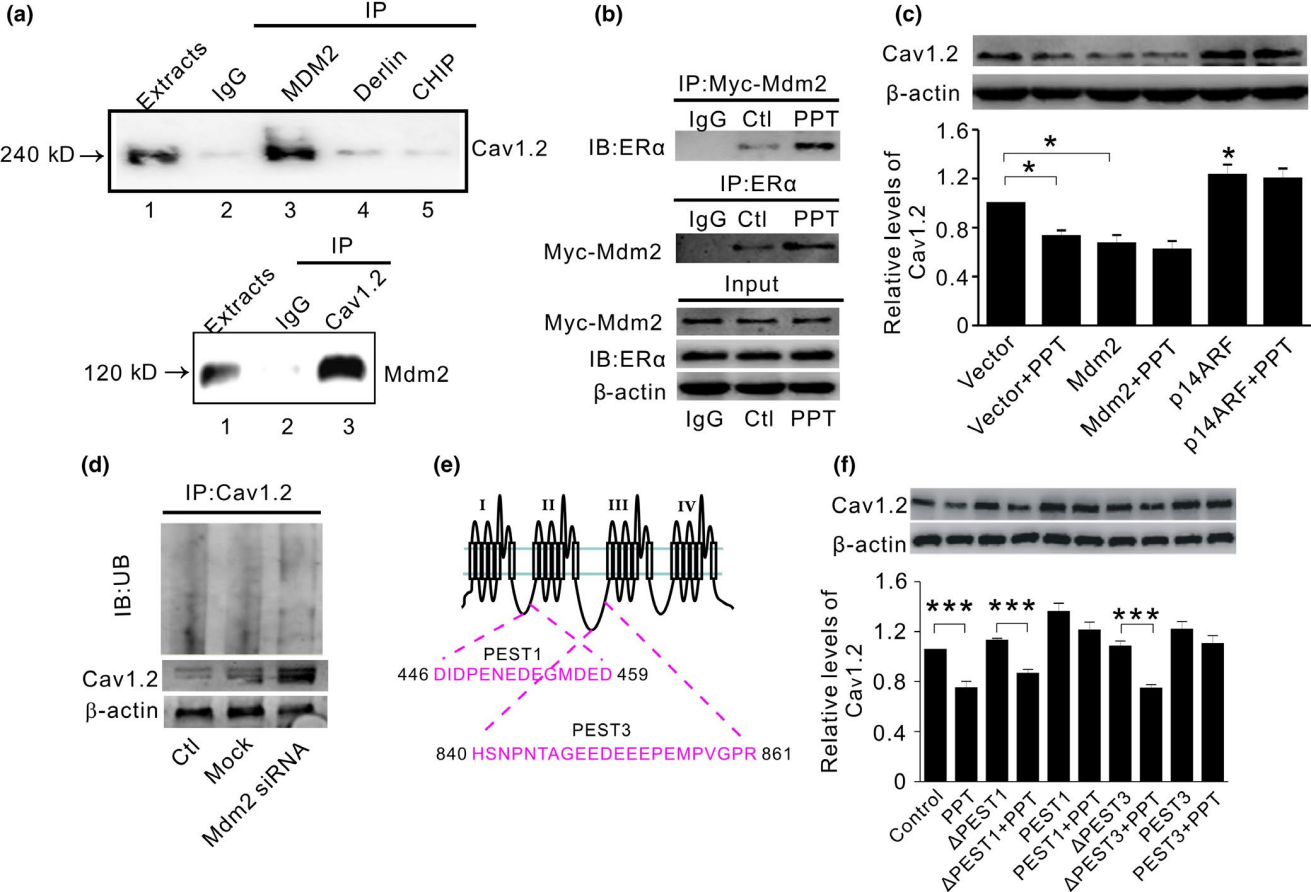
E3 ligase Mdm2 associated with PEST sequence of Cav1.2 is involved in ERα‐induced Cav1.2 degradation. (a) Representative Western blots of Cav1.2 (top) and Mdm2 (bottom) in HT22 cell extracts immunoprecipitated by control antibody IgG or antibodies against Mdm2, Derlin‐1, or CHIP (top), or by Cav1.2 antibody (bottom), respectively. Extract (lane 1) represents 10% of total protein used for immunoprecipitation. Mdm2 shows a strong association with Cav1.2. (b) Representative Western blots of ERα (top) and Mdm2 (middle) in HT22 cell extracts immunoprecipitated by control antibody IgG or antibodies against Mdm2 or ERα, respectively. Proteins in input are shown on the bottom. PPT treatment increases ERα association with Mdm2. (c) Representative Western blots (top) and quantification (bottom) of Cav1.2 in HT22 cells transiently expressing vector, Mdm2 or Mdm2 inhibitor p14ARF, in the absence and presence of PPT, respectively (*n* = 3). (d) Top: Western blots of ubiquitin in SH‐SY5Y cells without treatment (Ctl), or transiently transfected with mock siRNA (Mock) or Mdm2 siRNA, after cell extracts were immunoprecipitated by Cav1.2 antibody. Bottom: corresponding Western blots of Cav1.2 under these conditions. (e) Schematic diagram depicting PEST1 and PEST3 sequences located in Cav1.2 protein. (f) Representative Western blots (top) and quantification (bottom) of Cav1.2 in primary neurons treated with 150 μM synthesized PEST peptides (PEST1 and PEST3) or synthesized scrambled peptides (ΔPEST1 and ΔPEST3), in the absence or presence of PPT (*n* = 4).*p＜0.05,**p＜0.01, ANOVA*.* Mdm2: double minute 2 protein; p14ARF: p14 alternate open reading frame

The E3 ligases Mdm2 (double minute 2 protein) and CHIP (STIP1 homology and U‐Box‐containing protein 1, STUB1) are reportedly associated with ERα signaling (Fan, Park, & Nephew, 2005; Liu, Schwartz, & Brooks, 2000). To test whether Cav1.2 might interact with these E3 ligases, we first assessed Cav1.2 association with Mdm2, CHIP, and Derlin‐1; the latter has no known association with ERα. As shown in Figure 3a, Cav1.2 was detected only in Mdm2 immunoprecipitates in HT22 cells. We further showed that Mdm2 binding to ERα was increased in HT22 cell treated with PPT (Figure 3b). Overexpression of Mdm2 significantly decreased Cav1.2 protein and diminished Cav1.2 reduction by PPT (Figure 3c). Conversely, inhibition of Mdm2 by p14ARF (p14 alternate open reading frame, CDKN2A), which blocks Mdm2 interaction with substrate proteins (Agrawal, Yang, Murphy, & Agrawal, 2006), led to a significantly increased Cav1.2 and further attenuated the effect of PPT on Cav1.2 (Figure 3c). Moreover, in cells transiently transfected with Mdm2 siRNA, the reduced Cav1.2 ubiquitination was concomitant with the dramatic increase in Cav1.2 protein (Figure 3d). These results suggested that Mdm2‐associated ubiquitination was critical in ERα regulation of Cav1.2.

It is reported that a PEST motif is involved in Mdm2‐mediated degradation of PSD95 (postsynaptic density 95) (Colledge et al., 2003). Interestingly, two PEST sequences (PEST1/3) have been found in Cav1.2 (Michailidis et al., 2014). Thus, we assessed the effect of membrane‐permeable synthetic PEST1/3 peptides on ERα regulation of Cav1.2 (Figure 3e). Effective intracellular localization of these peptides and Cav1.2 enhancement were proved by fluorescent and Western blotting analyses (Figure S3). As shown in Figure 3f, compared with control or scrambled peptides (ΔPEST1/ΔPEST3), Cav1.2 was significantly increased in cells treated with PEST1/PEST3, in which PPT‐induced reduction of Cav1.2 was diminished. These results suggested that PEST motif in Cav1.2 played an important role in Mdm2‐mediated ubiquitination of Cav1.2.

**Figure 4:**
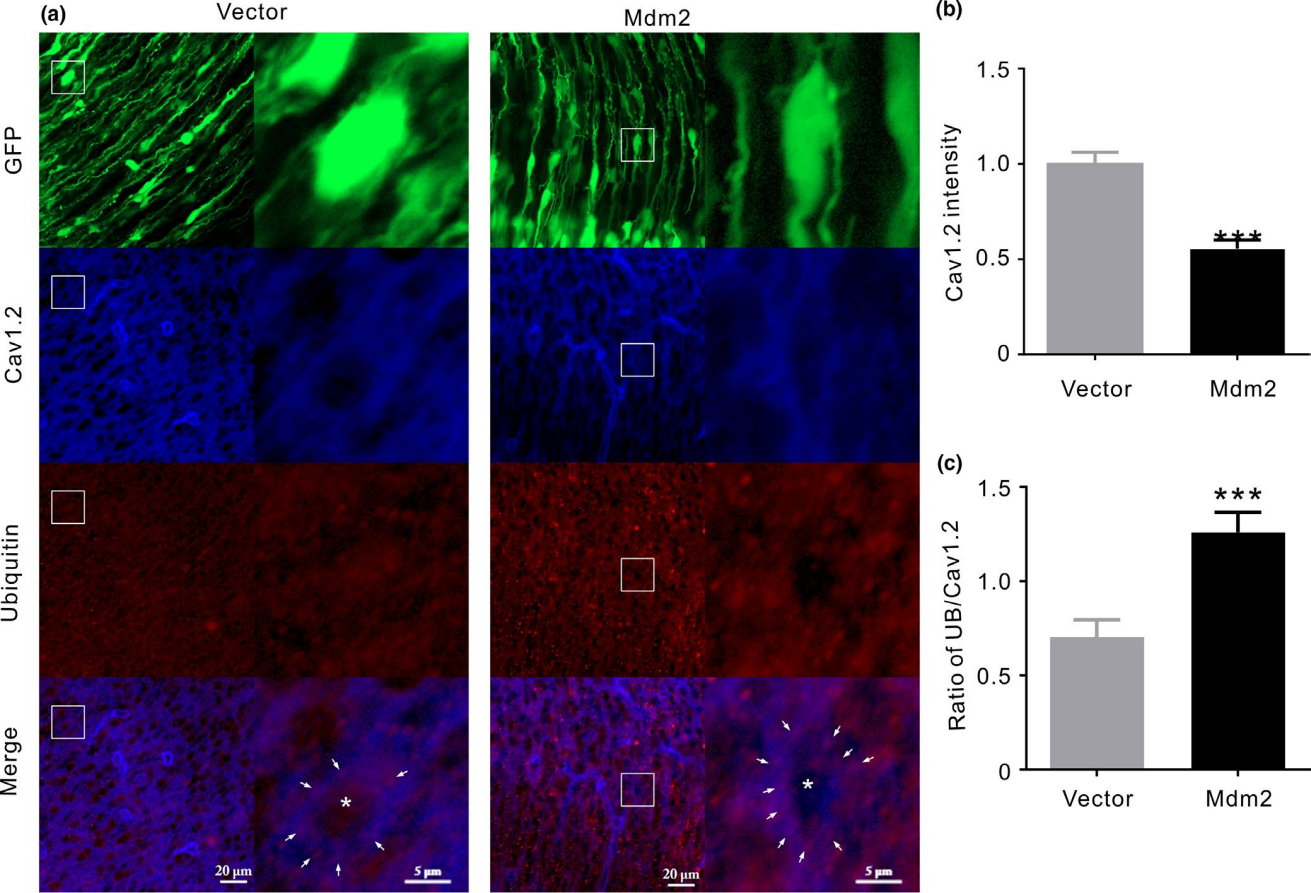
Mdm2 reduces Cav1.2 protein and enhances Cav1.2 ubiquitination in vivo. (a) Immunofluorescent images of cortical slices. Cells are successfully transfected with vector and Mdm2 as shown by green fluorescence (GFP). Cav1.2 and ubiquitin are shown as blue and red, respectively, whereas the merged images showing the colocalization of Cav1.2 and ubiquitin are shown as purple (Merge). The two columns under Vector or Mdm2 show general arrangement of cells (left) and the amplified view of immunofluorescence (right) from the rectangles marked in the left sides, respectively. The white arrows denote the purple dots indicating the colocalization of Cav1.2 and ubiquitin, whereas asterisk denotes the central area of cell body. (b) Quantitative analysis of Cav1.2 intensity between vector and Mdm2 (*n* = 36 cells/ 6 slices in each). (c) Quantitative analysis of ubiquitin intensity per Cav1.2 unit in cells transiently expressing vector or Mdm2 (*n* = 36 cells/ 6 slices in each). ***p＜0.001, unpaired *t* test

To further verify that Mdm2 affected Cav1.2 protein and ubiquitination in vivo, we introduced Mdm2 constructs into fetal brain using the in utero electroporation technique and assessed cortical neuronal Cav1.2 protein and its ubiquitination after birth by using immunofluorescent analysis. We found that in Mdm2‐overexpressing neurons (Figure 4a), the intensity of Cav1.2 was significantly decreased (Figure 4b), whereas the colocalization of ubiquitin and Cav1.2 was significantly increased, as measured by ubiquitin intensity per Cav1.2 unit (Figure 4c). These results indicated that Mdm2 facilitated Cav1.2 ubiquitination and degradation in vivo.

### 2.4 PPT treatment reduced Cav1.2 protein in OVX APP/PS1 mice

**Figure 5:**
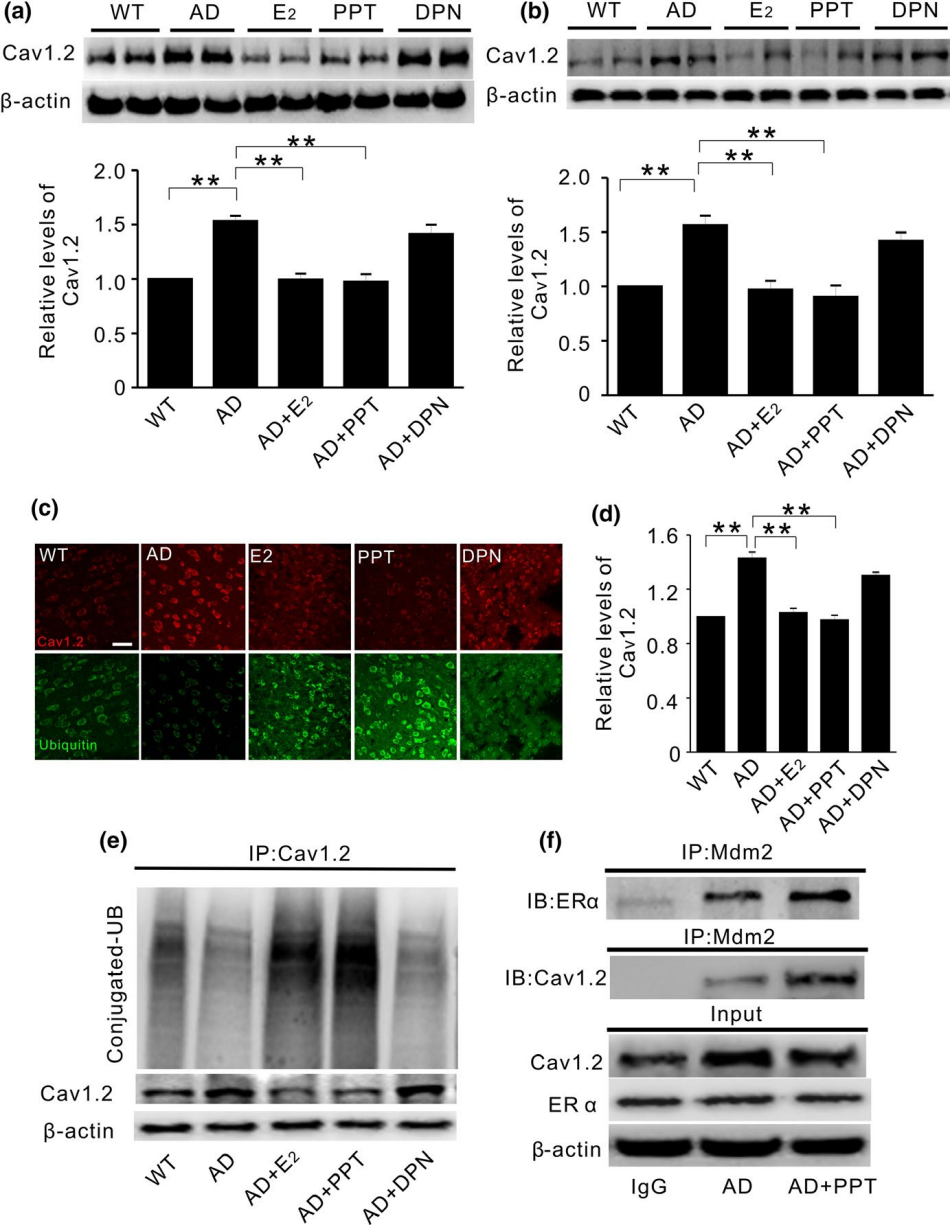
PPT treatment reduces Cav1.2 protein in ovariectomized APP/PS1 mice. (a and b) Representative Western blots (top) and quantification (bottom) of Cav1.2 in the hippocampus (a) and the cortex (b) of ovariectomized wild‐type mice without any treatment (WT, *n* = 8), or ovariectomized APP/PS mice treated with vehicle (AD, *n* = 7), 17β‐estradiol (E2, 30 μg/kg, *n* = 8), PPT (1 mg/kg, *n* = 8), and DPN (1 mg/kg, *n* = 7) for two weeks. E2 or PPT replacement attenuates Cav1.2 elevation in OVX APP/PS1 mice. (c) Representative immunofluorescent images from cortical slices probed with anti‐Cav1.2 (red) and anti‐ubiquitin (green) antibodies, in WT or OVX APP/PS1 mice. (d) Quantification of Cav1.2 immunofluorescent density using data related to C. (e) Western blots of ubiquitin (UB) in cortical extracts immunoprecipitated by Cav1.2 antibody, showing that Cav1.2 ubiquitination is significantly increased in APP/PS1 mice treated with E2 or PPT, but not with DPN. (f) Western blots of ERα (top) and Mdm2 (middle) in the hippocampal extracts of OVX APP/PS1 mice immunoprecipitated by Mdm2 antibody. Proteins in input are shown on the bottom. PPT treatment increases Mdm2 association with ERα and Cav1.2. *p＜0.05, **p＜0.01, ***p＜0.001, ANOVA

As shown in Figure 5a,b, the basal level of Cav1.2 level was significantly increased in the hippocampus and cortex of OVX APP/PS1 (AD) mice compared to WT mice. The elevated Cav1.2 was reversed by E2 or PPT, but not by DPN. Accordingly, while Cav1.2 association with UB was significantly decreased in AD (AD vs. WT), an increased ubiquitination of Cav1.2 was seen in E2 (AD + E2)‐ or PPT (AD + PPT)‐treated AD mice but not DPN (DPN + AD)‐treated AD mice (Figure 5c–e). Moreover, Mdm2 association with Cav1.2 or ERα was increased in mice treated with PPT, respectively (Figure 5f). These results indicated that ERα activation promoted the ubiquitination and degradation of Cav1.2 in OVX APP/PS1 mice.

### 2.5 PPT treatment attenuated cognitive decline in APP/PS1 mice

**Figure 6:**
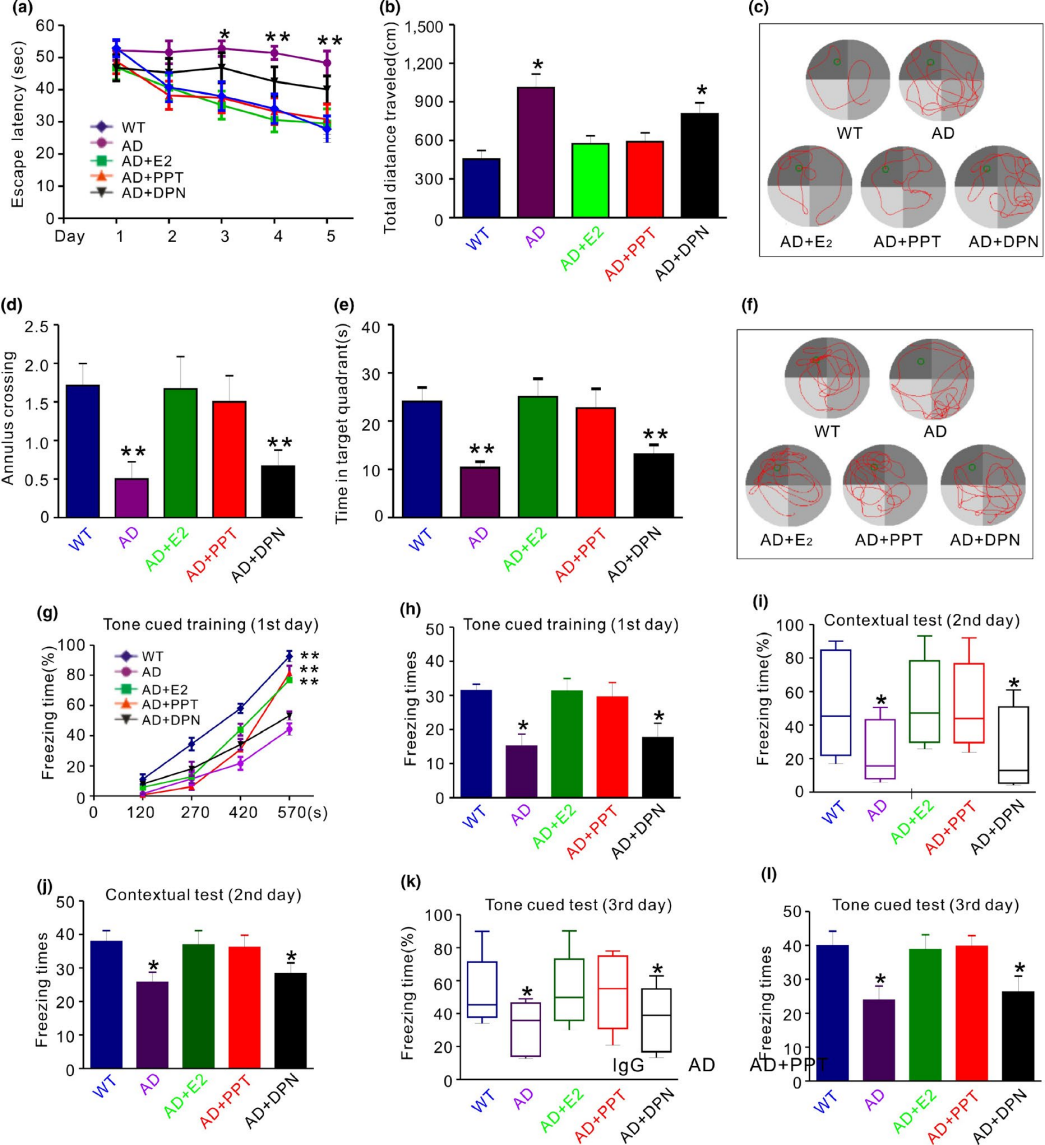
PPT treatment improves spatial and associative learning and memory performance in OVX APP/PS1 mice. (a) Compared with vehicle‐treated APP/PS1 mice (AD), E2 (AD + E2)‐ or PPT (AD + PPT)‐treated APP/PS1 mice show a significantly shorter latency on the third, fourth, and fifth day, respectively. No significant difference is found between DPN‐treated mice and AD mice. (b) In the hidden platform tests on the fifth day, the total distance spent on reaching the platform was recorded. Compared with vehicle‐treated APP/PS1 mice, PPT‐ or E2‐treated APP/PS1 mice show a significantly shorter distance. No significant difference is found between DPN‐treated mice and AD mice. (c) Representative road maps showing the movement trajectory of mice in hidden platform experiment on the fifth day. (d and e) In the probe trial on the sixth day, PPT‐ and E2‐treated mice show significantly more times traveling (annulus crossing, d) and significantly longer time staying (e) in the place where the hidden platform was previously placed, compared with AD. (f) Representative road maps showing the movement trajectory of mice in the probe trial on the sixth day. (g and h) Percentage freezing time as a function of training periods (g) and freezing times (H) are shown in wild‐type mice (WT), APP/PS1 mice treated with vehicle (AD), E2 (AD + E2), PPT (AD + PPT), and DPN (AD + DPN), respectively, on the first day. (i–l) On the second day (I and J) and third day (K AND L), percentage freezing time (i and k) and freezing times (j and l) are shown in the five groups of mice. Compared with AD + E2 or AD + PPT, mice in AD and AD + DPN exhibit significantly reduced freezing time and times of freezing at all points tested. No significance is shown between AD and AD + DPN. *p < 0.05, **p < 0.01 (ANOVA, *n* = 7–8 per group)

We next assessed the effect of E2 or ER agonists on spatial and associative learning and memory performances in OVX APP/PS1 mice. In the hidden platform test, the escape latency and total traveling distance in E2‐ or PPT‐treated OVX APP/PS1 (AD + E2 or AD + PPT) mice were significantly shorter than that in saline‐treated OVX APP/PS1 (AD) mice beginning on the third day (Figure 6a–c). In the probe trial when the platform was removed, the passing times crossing over target site and the staying time in target quadrant were significantly more in AD + E2 and AD + PPT than those in AD or AD + DPN mice (Figure 6d–f). The subsequent contextual fear conditioning tests revealed that AD + E2 and AD + PPT mice exhibited more percentage freezing time (Figure 6g) and the longer time of freezing than AD and AD + DPN on the first day (Figure 6h), respectively. The second and third day tests revealed the similar results (Figure 6i‐l). These results indicated that PPT significantly improved spatial and associative learning memory in OVX APP/PS1 mice.

## 3 DISCUSSION

The efficacy of estrogen‐based hormone therapy (HT) in AD is not conclusive. Some studies reveal that HT may reduce the risk of AD in postmenopausal women (Zandi et al., 2002), whereas others find that HT does not reduce dementia or even has an adverse effect (Shumaker et al., 2004). One explanation is that compared with the natural E2, the conjugated equine estrogen used in clinics does not easily diffuse into the brain (Lan, Zhao, & Li, 2015; Steingold, Cefalu, Pardridge, Judd, & Chaudhuri, 1986). Another explanation is that the initiation of the therapy may have been delayed (Sherwin, 2005). Given that LTCC blockers attenuate AD‐associated pathology, E2‐induced reduction of Cav1.2 in our study favors the beneficial role in AD.

Evidence has suggested that UPS is involved in Cav1.2 degradation. For instance, the ubiquitin ligase RFP2 (Ret finger protein 2) mediates proteasomal degradation of Cav1.2 in neuronal cells (Altier et al., 2011). Cav1.2 degradation is promoted by ubiquitin ligase NEDD4‐1 (neuronal precursor cell‐expressed developmentally down‐regulated 4‐1) and is inhibited by deubiquitinase USP2‐45 (ubiquitin‐specific protease 2‐45) in kidney cells (Rougier, Albesa, Abriel, & Viard, 2011; Rougier et al., 2015). The calcium‐dependent cleavage of Cav1.2 fragment channels through UPS increases with aging, which can be blocked by LTCC inhibitor in vivo (Michailidis et al., 2014). Consistently, proteasomal inhibitor MG132 prevents Cav1.2 degradation by ERα (Figure 2), suggesting that ERα may use UPS to monitor Cav1.2 degradation.

The seven lysine residues in UB play distinct roles in cellular function (Swatek & Komander, 2016). While K29‐linked chains and K29‐specific deubiquitinating enzyme TRABID are involved in proteasome regulation (Kim et al., 2011; Kristariyanto et al., 2015), K6‐linked UB chains are associated with mitophagy but with no known functions in UPS (Swatek & Komander, 2016). We found that K6‐ or K29‐overexpressing cells exhibit significantly decreased Cav1.2 under basal condition. However, K6R or K29R mutant alone did not alter Cav1.2 (Figure 2g). The underlying mechanisms are currently not well‐understood. One explanation may be that K6/ K29 differs from K6R/ K29R in the existence of other Ks in UB constructs. In K6‐/ K29‐expressing cells, when other Ks were mutated, the presence of K6/ K29 might help the ubiquitination and degradation of Cav1.2. In contrast, in K6R‐ or K29R‐overexpressing cells, the other Ks are not mutated and might be functional. In K29R for instance, the existence of K6 might compensate for the effect of K29R. It seems that a combination of K6R and K29R is sufficient to increase Cav1.2 protein (Figure 2g), suggesting that the poly‐UB chains containing K6 and K29 are important in Cav1.2 degradation. In line with this, in cells transfected with UB mutants other than K6/ K29 including K11, K27, K33, K48, and K63, when both K6 and K29 were among those being mutated, the increase of Cav1.2 occurs in control condition (Figure 2e). Interestingly, only K29 seems to be critical in ERα regulation of Cav1.2, as PPT‐induced reduction of Cav1.2 is diminished only in K29R but not in K6R‐overexpressing cells. In line with this, the deubiquitinating enzyme TRABID that selectively acts on K29 in mammalian cells also diminishes PPT effect (Figure 2i).

ERα is known to physically interact with Mdm2 (Liu et al., 2000), which mediates ubiquitination through K11‐, K29‐, and K63‐linked UB polymers (Wu & Leng, 2015). In addition, the conserved PEST motif is involved in Mdm2‐mediated target protein degradation (Colledge et al., 2003). It has been reported that similar PEST sequences are located in Cav1.2, which provides signaling for UPS in calcium‐induced mid‐channel proteolysis and Akt‐induced Cav1.2 degradation (Catalucci et al., 2009; Michailidis et al., 2014). In our study, Mdm2 involvement in ERα regulation of Cav1.2 might have been supported by the following findings: (a) Mdm2 reduces Cav1.2 protein and increases its ubiquitination (Figure 4). (b) Mdm2 association with ERα is promoted by PPT treatment (Figure 3b), which also increases Mdm2 association with Cav1.2 (Figure 5f). (c) Mdm2 overexpression or inhibition diminishes PPT effect on Cav1.2 (Figure 3c). (d) The synthesized PEST sequences, which is known to play a role in Mdm2‐associated protein degradation (Colledge et al., 2003), attenuate PPT‐induced reduction of Cav1.2 (Figure 3f).

In physiological conditions, both ERα and ERβ seem to be involved in spatial memory enhancement (Walf, Paris, & Frye, 2009). ERα agonist PPT or ERβ agonist DPN improves cognitive function in OVX mice and rats (Boulware, Heisler, & Frick, 2013; Hammond, Mauk, Ninaci, Nelson, & Gibbs, 2009). In AD however, only ERα shows beneficial role (Lan et al., 2015). PPT but not DPN rescues cognitive decline and Aβ deposition in another AD model 3xTg‐AD mice (Carroll & Pike, 2008). This inconsistency may be associated with different neuroprotective effects of PPT and DPN in pathological conditions. While PPT prevents Aβ‐induced neuronal death in hippocampal neurons (Merlo, Spampinato, Capani, & Sortino, 2016), DPN is modestly protective (Thibault et al., 2007). In our study, PPT‐induced Cav1.2 reduction may suggest an important role of LTCC in estrogen‐mediated neuroprotection. It is reported that LTCC mediates Aβ‐induced calcium entry, which in turn facilitates further Aβ formation (Demuro, Parker, & Stutzmann, 2010). Conversely, selective LTCC blocker rescues cortical neurons from Aβ‐induced cell death and improves cognitive function in AD (Nimmrich & Eckert, 2013). It may be reasonable to speculate that Cav1.2 reduction and the associated neuroprotection play a role in ERα‐induced cognitive improvement in APP/PS1 mice.

We propose possible mechanisms through which ERα reduces Cav1.2 protein (Figure S4). Activation of ERα (by PPT) facilitates Mdm2 association with Cav1.2. This process involves K29‐linked UB chains and PEST motif in Cav1.2, which may provide a signal for subsequent proteasomal degradation. In APP/PS1 mice, the reduced Cav1.2 protein may be involved in ERα‐induced cognitive improvement. Meanwhile, it is worth noting that all estrogen‐based manipulations are limited to only AD model in our study. In the course of aging, which is different from AD in pathological biomarkers and clinical manifestations, whether estrogen may regulate Cav1.2 through similar ubiquitination mechanisms described here requires further investigation.

## 4 EXPERIMENTAL PROCEDURES

### 4.1 Antibodies and reagents

Antibodies against Cav1.2 (ab84814 and ACC‐003) were from Abcam (Cambridge, UK) and Alomone, respectively, which produced identical Western blots. Those against β‐actin (ab6276), MAP‐2 (ab32454), ERα (ab3573), ERβ (ab104804), calcineurin (ab3673), Mdm2 (ab16895), and ubiquitin (ab19247) were purchased from Abcam. pSer1928‐Cav1.2 (A010‐70) was from Badrilla (United Kingdom). 17β‐estradiol (E2758), propylpyrazoletriol (PPT, H6036), diarylprepionitrile (DPN, H5915), E2‐BSA (E5630), ICI 182,780 (V900926), nifedipine (N7630), FK506 (F4679), MG132 (C2211), ammonium chloride (A0171), chloroquine (C6628), carfilzomib (791938), and other reagents were purchased from Sigma (St. Louis, MO, USA). Lipid solvents were made in stock solutions in DMSO (1:1,000 ~ 2,000).

### 4.2 Gene constructs

pCMV6‐Myc‐Mdm2 and pCMV6‐Myc‐p14ARF were purchased from OriGene (USA). TRABID (DU# 49067) and TRABID mutant C443A (DU# 49089) were purchased from MRC PPU Reagents and Services (University of Dundee). pRK5‐HA‐ubiquitin was obtained from Addgene. All ubiquitin mutants were made from pRK5‐HA‐ubiquitin plasmid by mutagenesis. pRK5‐HA‐Ubiquitin‐K0 (no lysine), pRK5‐HA‐Ubiquitin‐K6 (keep lysine at position 6), pRK5‐HA‐Ubiquitin‐K11, pRK5‐HA‐Ubiquitin‐K27, pRK5‐HA‐Ubiquitin‐K29, pRK5‐HA‐Ubiquitin‐K33, pRK5‐HA‐Ubiquitin‐K48, pRK5‐HA‐Ubiquitin‐K63, pRK5‐HA‐Ubiquitin‐K6R (keep all lysine residues except lysine at position 6 mutated to arginine), pRK5‐HA‐Ubiquitin‐K29R, pRK5‐HA‐Ubiquitin‐K63R, CHIP with myc tag, and Derlin‐1 with HA tag were cloned to pcDNA3, respectively.

### 4.3 Primary culture of cortical neurons

Cortical neurons were extracted from prenatal pups on embryonic day 18. Cells were placed on poly‐L‐lysine (0.1 mg/ml)‐coated 6‐well plates, at a density of 2 × 10^6 ^cells/ml for biochemical experiments and 0.5 × 10^6 ^cells/ml for molecular and immunohistochemistry experiments. Cultures were maintained in serum‐free neurobasal (NB) medium (2% B27, 2 mM glutamine, 1% penicillin/streptomycin; Invitrogen) at 37°C with 5% CO_2_. Fifty percent of the medium was exchanged with fresh medium every other day for 2 weeks. On the day before experiment, culture medium was completely replaced by fresh medium in the presence of accurate dilution of chemicals.

### 4.4 Cell culture and gene transfection

HT22 (immortalized mouse hippocampal) and SH‐SY5Y (human neuroblastoma) cell lines were obtained from the Type Culture Collection of the Chinese Academy of Sciences (Shanghai, China). Cells were incubated in Dulbecco's modified Eagle's medium (DMEM, Invitrogen) containing 4.5 g glucose/L, supplemented with penicillin/streptomycin (50 units/mL), glutamate (2 mM), and 10% fetal bovine serum (FBS, Gibco), and were maintained at 37°C with 10% CO_2_. At ~ 90% confluence, cells were transiently transfected with various plasmids or Mdm2 siRNA using the Lipofectamine 2000 reagent (Invitrogen) according to the manufacturer's instructions. A total of 2.5 μg of DNA in 100 μl Opti‐MEM (Invitrogen) were used in each of six wells. The following human Mdm2 siRNA sequences were used for transfection in SH‐SY5Y cells: sense CGUACGCGGAAUACUUCGATT, antisense AAUCGAAGUAUUCCGCGUACG; NC: sense CGUACGCGGAAUACUUCGATT, antisense AAUCGAAGUAUUCCGCGUACG (100 pM per six wells for 72 hr). The effectiveness of Mdm2 knockdown was verified by Western blotting analysis.

### 4.5 Western blotting and co‐immunoprecipitation

Western blotting of Cav1.2 was performed as previously described (He et al., 2016). Blots were visualized with chemiluminescence detection reagent kit (Millipore, 345818) and captured with a Fusion chemiluminescent imaging system (Vilber Lourmat, France). Signal intensities from each band were quantified by Quantity One software.

For co‐immunoprecipitation (Co‐IP) assays, the cell or tissue extracts (0.5 mg) were pre‐incubated with protein A agarose beads (Beyotime Biotechnology, P2012) for 3 hr to reduce nonspecific reaction. The supernatants were then mixed with nonspecific IgG and appropriate antibodies for 1 hr. The mixtures were then incubated with protein A agarose beads (Beyotime Biotechnology, P2012) overnight at 4°C. Samples were washed two times with lysis buffer and two times with sterile PBS and denatured with SDS sampling buffer.

### 4.6 Immunofluorescence

Brain sections were washed with ice‐cold PBS, then fixed in ice‐cold 4% paraformaldehyde for 10 min, permeabilized in 0.1% Triton X‐100 for 10 min, and blocked with 10% normal goat serum for 1 hr at 37°C with extensive PBS washings between each step. Specimens were incubated with appropriate primary antibodies (1:50 in PBS) at 4°C overnight. The sections were then washed 6 × 2 min with PBS, followed by incubation with the secondary antibody Alexa 549 or Fluro 488 (1:100 in PBS) at 37°C for 1 hr. After sealing with a mixture of glycerol and PBS at a ratio of 1:1, the immunofluorescent‐labeled sections were examined using a laser scanning confocal microscope (Nikon, Japan) with an Olympus IX 70 inverted microscope (Olympus, Tokyo, Japan) equipped with a Fluoview FVX confocal scan head.

### 4.7 Whole‐cell patch‐clamp recording

Voltage‐dependent calcium‐mediated currents (ICa^2+^) were recorded from cultured cortical neurons from 7 to 11 days in vitro (DIV), at room temperature using a MultiClamp 700B amplifier (Axon Instruments). The recording chamber was perfused with Tyrode's solution containing (in mM): NaCl 100, tetraethylammonium‐Cl 20, 4‐AP 5, KCl 4, MgCl_2_ 1, BaCl_2_10, glycine 0.01, HEPES 25, glucose 30, and TTX 0.001. The glass micropipettes had a resistance of 3–5 MΩ when filled with internal solution containing (in mM): CsCl 135, MgCl_2_ 4, HEPES 10, EGTA 10, MgATP 4, and Na_3_GTP 0.3. A prepulse protocol consists of 300–700 ms to −30 mV followed by 50 ms to −50 mV, before each test pulse was used to inactivate T‐type Ca^2+^ channels and Na+ channels. ICa^2+^ was elicited by 300 ms test pulses of variable amplitude (−40 to +60 mV at a step of 10 mV) from a holding potential of −60 mV. The interval between test pulses was 10 s.

### 4.8 Quantitative RT–PCR

RT–PCRs were performed on a Bio‐Rad IQ5 detection system (Bio‐Rad, Hercules, CA, USA). The primer sequences for Cav1.2 were as follows: 5′‐TACCGTCAGTTCCACACAGC‐3′ and 5′‐CTTCAGAGTCAGGCAGAGCA‐3′. The threshold cycle (Ct) value of each sample was calculated, and the relative mRNA level was normalized to the GAPDH mRNA value.

### 4.9 Lentivirus** infection**

The target shRNAs against rat ERα/β gene (NM_012689/NM_012754) were designed as follows: ERα‐1: GATAAGAACCGGAGGAAGA, ERα‐2: CCAGAATGGCCGAGAGAGA, and ERα‐3: GTAAATGTGTAGAAGGCAT; ERβ‐1: TGAGCAAAGCCAAGAGAAA, ERβ‐2: TCACTAAGCTGGCGGACAA, and ERβ‐3: CCAAATGTGCTATGGCCAA. Negative control was the scrambled sequence with no homology to rat genes. The recombinant lentiviral vector bearing rat *ERα/β* shRNAs was produced by Shanghai GeneChem Co., Ltd. (Shanghai, China). At 10 DIV, ER‐related lentivirus was added to the culture for 72 hr according to the manufacturers’ instruction (GeneChem, Shanghai, China), while transfection of Mdm2 was performed in HT22 cells.

### 4.10 In utero electroporation and related immunofluorescence

Briefly, *in utero* electroporation was performed on pregnant C57/B6L mice on embryonic day 14.5 (E14.5) as previously described (Saito, 2006). The vector and Mdm2 plasmids were mixed with the Venus‐GFP with a ratio of 3:1 at a final concentration of 1,000 ng/μl. The mixture was injected into lateral ventricle. The electrode (CUY650P5, NEPA GENE) was positioned flanking the ventricular zones with the anode on the target side, and the embryos were then pulsed with a super electroporator NEPA21 type II (NEPA GENE) according to the following procedure: voltage = 33 V, pulse length = 50 ms, pulse interval = 950 ms, pulse number = 3. The uterus was then returned to the abdominal cavity for further development. After birth, the brains were perfused with PBS on P0 and were fixed in 4% PFA (Sigma) overnight at 4°C, and then were replaced with 20% sucrose for 8 hr, followed by 30% sucrose at 4°C for an additional 8 hr. The brains were cut into coronal slices using a cryostat microtome (CM1950, Leica) and subjected to immunofluorescence staining. Slices were permeabilized and blocked in 10% goat serum, 3% BSA, and 0.3% Triton X‐100 in PBS. Brain sections were then incubated with primary antibody (Cav1.2 1:100, Ubiquitin 1:100) for 1 hr at 37°C and then with the fluorescent‐conjugated secondary antibodies (goat anti‐mouse Alexa 647 1:100, goat anti‐rabbit Cy3 1:100) for 1 hr at 37°C. Confocal images were acquired and analyzed with the ZEISS system (LSM800). All animal studies were approved by the animal care committee of Chongqing Medical University. Image assay was performed using ImagePro Plus 6.0.

### 4.11 Peptides synthesis and treatment

Peptide sequences that were designed to mimic the PEST1 (S446/459) and PEST3 (S840/861) were synthesized and purified by Pepmic Co. Ltd (Suzhou, China). The sequences were as follows: YGRKKRRQRRR‐DIDPENEDEGMDED‐GFP for TAT‐PEST1, YGRKKRRQRRR‐DIDKENEDEGKDED‐GFP (ΔPEST1) for scrambled, YGRKKRRQRRR‐HSNPNTAGEEDEEEPEMPVGPR‐GFP for TAT‐PEST3, and YGRKKRRQRRR‐HSNPNPAGEEDKEEPAMPVGPR‐GFP (ΔPEST3) for scrabbled control (Yu, Wu, Liu, Ge, & Wang, 2008). They were all directly diluted in serum‐free neurobasal (NB) medium before use. At 9 DIV, primary neurons were washed once and maintained in antibiotic‐free NB medium overnight. Cells were subsequently treated with 0, 5, 100, or 150ìM PEST peptides for 0, 2, 6, and 12 hr at 37°C with 5% CO2, respectively.

### 4.12 Animal treatment

Wild‐type and APPswe/PS1E9 transgenic mice (APP/PS1) were obtained from Nanjing University (China). Eight‐month‐old female APP/PS1 mice were bilaterally ovariectomized (OVX) for 14 days. Animals were subcutaneously injected daily with vehicle, 17β‐estradiol (E2, 30 μg/kg), PPT (1 mg/kg), and DPN (1 mg/kg) for two weeks. The dosage of E2 is thought to be within physiological range; and those of PPT and DPN are functional (Avtanski et al., 2014).

### 4.13 Morris water maze and contextual and cued fear conditioning

The Morris water maze test included four platform trials per day for five consecutive days and a probe trial on the sixth day. Swimming activities (latency, distance, and strategy of search) were recorded by a video and analyzed by image analyzing software (ANY‐maze; Stoelting). For contextual and cued fear conditioning, mice were tested in a 3‐day paradigm. Behavior was recorded by video camera, and freezing data were measured using FreezeScan software.

### 4.14 Statistical analyses

Data were presented as means ± *SEM* from at least three independent experiments. The statistical comparisons between two groups were tested using Student's *t* test. The comparisons among groups were tested using one‐way or two‐way ANOVA, and a post hoc pairwise comparison was used where it applied.

## CONFLICT OF INTEREST

None declared.

## AUTHOR'S CONTRIBUTIONS

G‐J Chen and Z Yan designed the research; Y‐J Lai performed the research and analyzed the data; B‐L Zhu, F Sun, D Luo, Y‐L Ma, B‐Luo, J Tang, M‐J Xiong, L Liu, X‐T Hu, L He, X‐J Deng, J‐H Zhang, and J Yang provided assistance with the research; G‐J Chen and Y‐J Lai wrote the paper. All authors read and approved the final manuscript.
