# Supplementary figures and images for: Estrogen receptor α promotes Cav1.2 ubiquitination and degradation in neuronal cells and in APP/PS1 mice
Source: Aging Cell. 2019 Apr 22;18(4):e12961. doi: 10.1111/acel.12961 (PMC6612642; doi:10.1111/acel.12961)

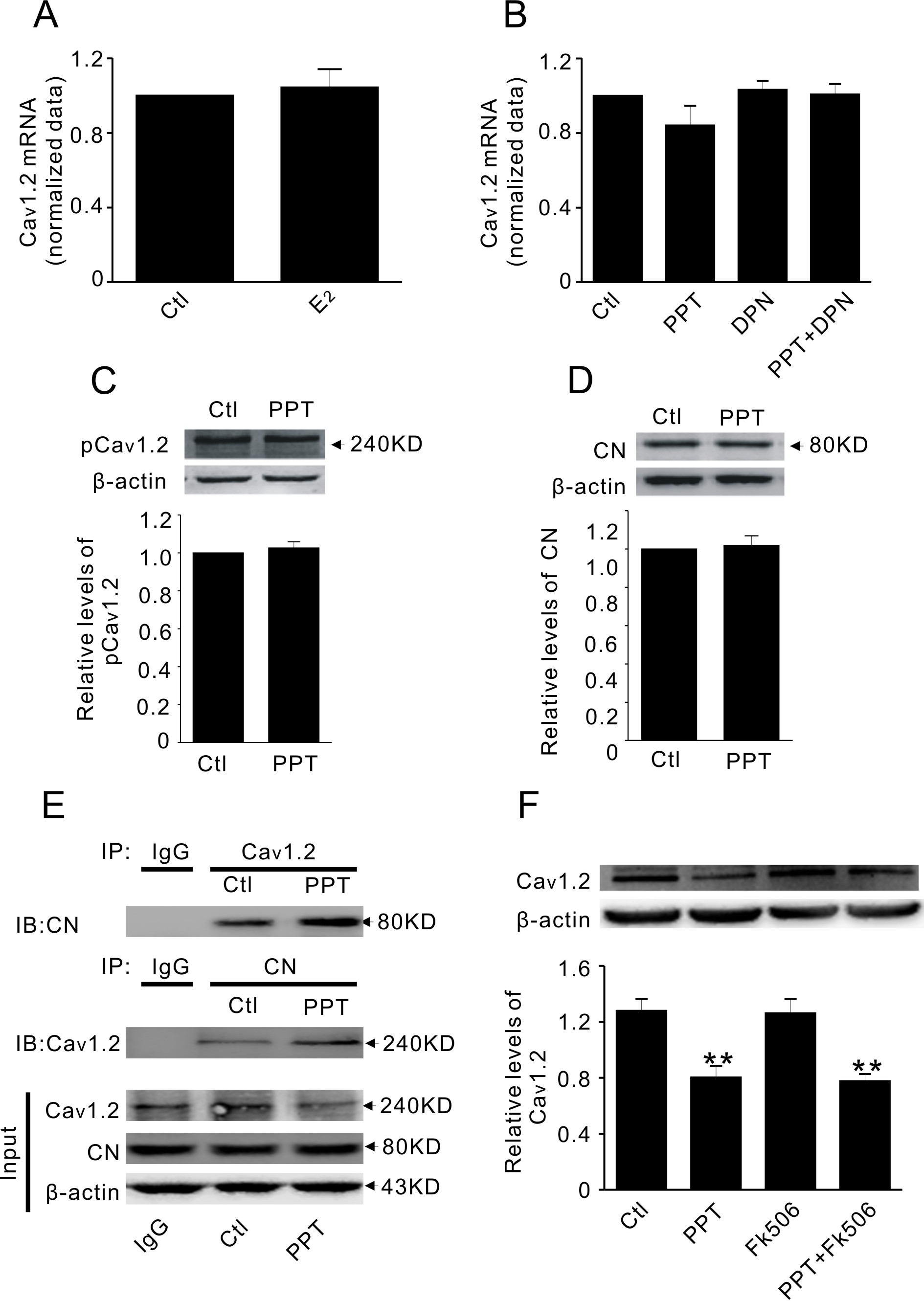

Supplement: Supplementary file 1 [file ACEL-18-e12961-s001.jpg]

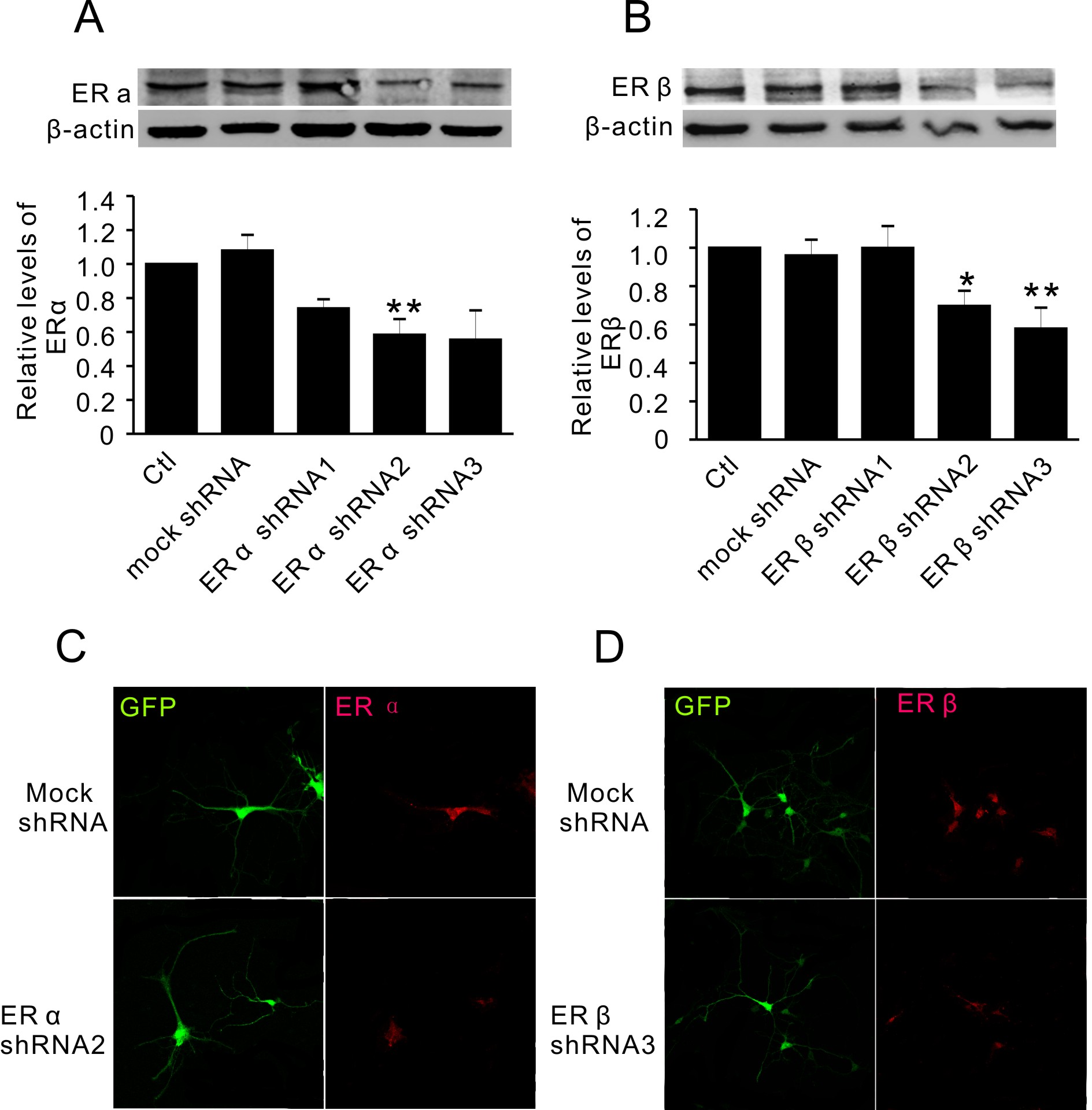

Supplement: Supplementary file 2 [file ACEL-18-e12961-s002.jpg]

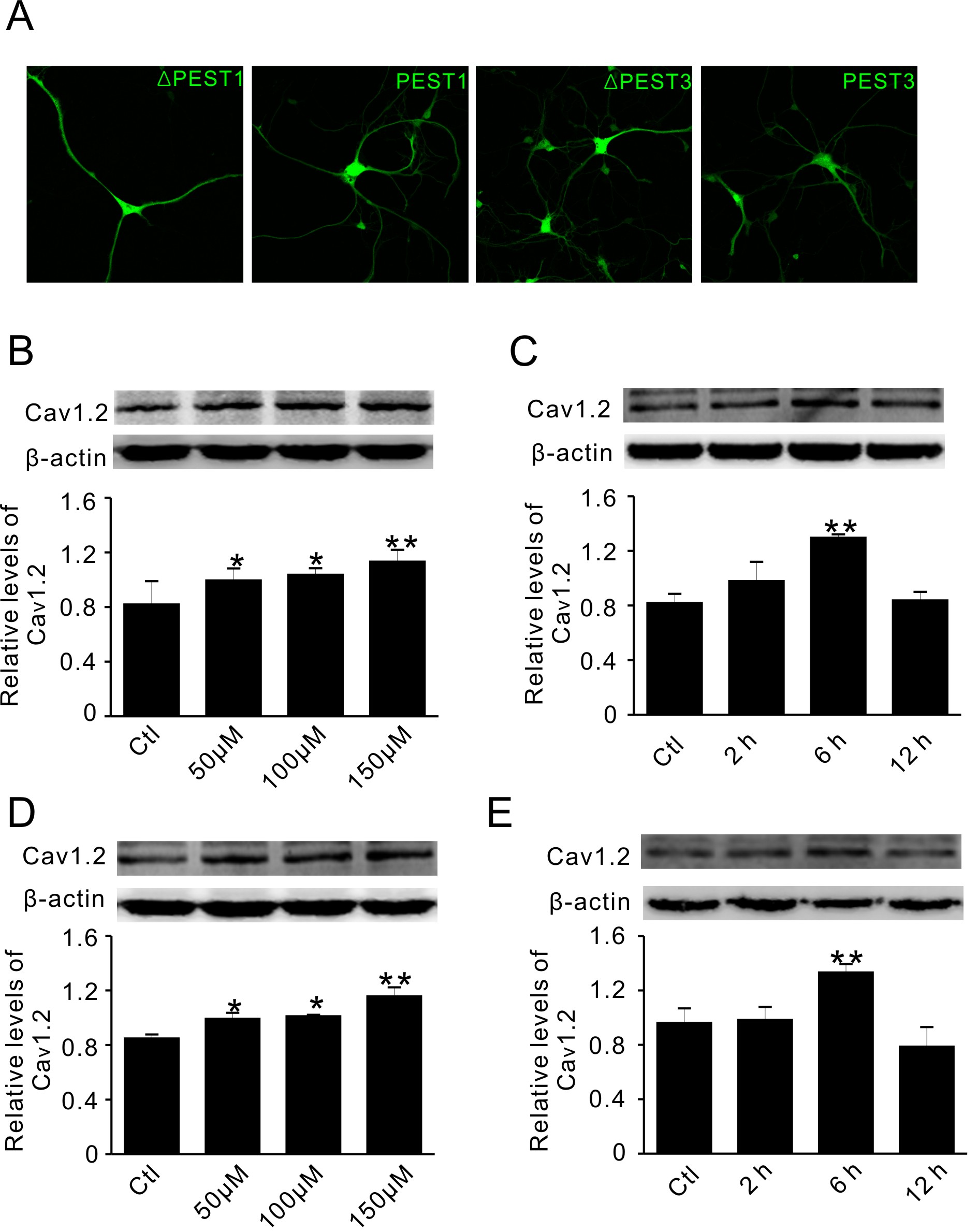

Supplement: Supplementary file 3 [file ACEL-18-e12961-s003.jpg]

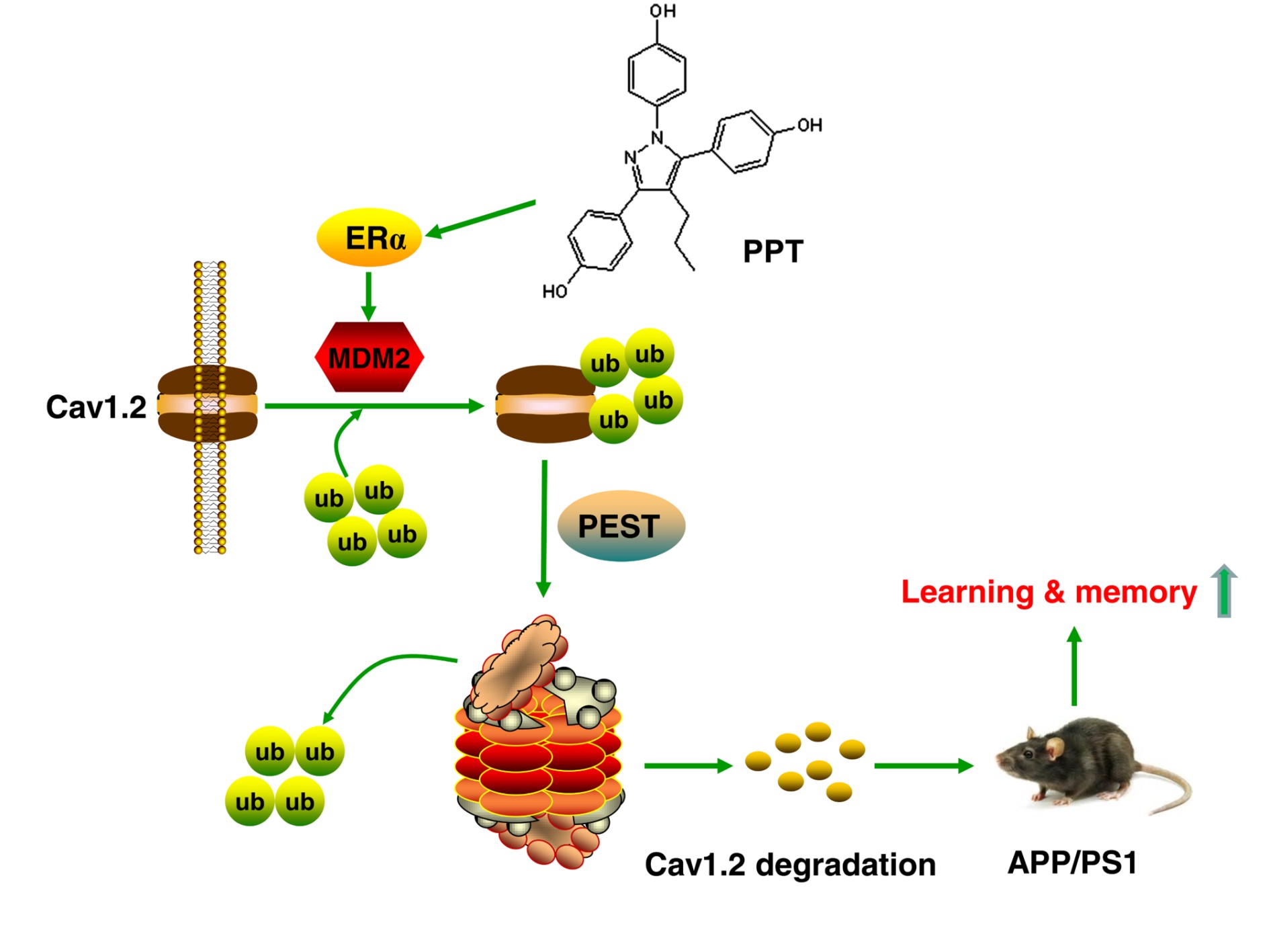

Supplement: Supplementary file 4 [file ACEL-18-e12961-s004.jpg]
