# Supplementary material for: Estrogen receptor α promotes Cav1.2 ubiquitination and degradation in neuronal cells and in APP/PS1 mice
Source: Aging Cell. 2019 Apr 22;18(4):e12961. doi: 10.1111/acel.12961 (PMC6612642; doi:10.1111/acel.12961)
Supplement: Supplementary file 2 [file ACEL-18-e12961-s005.odt]

Supplemental Figure legends

Figure S1：ERα / ERβ shRNA effectively reduces target protein in primary cortical neurons. (A) Representative Western blots (top) and quantification (bottom) of ERα in neurons transfected with mock shRNA or ERα shRNAs (ERα shRNA1-3). ** P < 0.01, ANOVA, n = 3. (B) Representative Western blots (top) and quantification (bottom) of ERβ in neurons transfected with mock shRNA or ERβ shRNAs (ERβ shRNA1-3). (C&D) Immunofluorescent images show effective knockdown of ERα(C) and ERβ (D) in primary cortical neurons transfected with the shRNA indicated. Red: ERα (C) or ERβ (D); Green: GFP. * P < 0.05, ** P < 0.01, ANOVA, n = 4. 

Figure S2. ERα regulation of Cav1.2 does not involve Cav1.2 gene transcription or protein phosphorylation. (A&B) Relative levels of Cav1.2 mRNA in primary cortical neurons in the absence (Ctl) or presence of E2 (A, n = 11 in each) and PPT, DPN and PPT+DPN (B, n = 3 in each). Transcripts of Cav1.2 are not significantly altered in any of these groups. (C&D) Representative Western blots (top) and quantification (bottom) of the phosphorylated Cav1.2 at Ser1928 (pCav1.2, C, n = 11) and calcineurin (CN, D, n = 9) in primary cortical neurons treated with PPT. (E) Co-immunoprecipitation images show that PPT increases Cav1.2 association with CN (top and middle). Western blots show that in the presence of PPT, Cav1.2 is significantly decreased while CN protein remains unchanged (bottom). (F) Representative Western blots (top) and quantification (bottom) of Cav1.2 in primary cortical neurons treated with vehicle (Ctl), PPT, CN inhibitor FK506 (10nM for 24 h) and PPT with FK506 (PPT+FK506). FK506 does not alter basal level of Cav1.2 and fails to abolish PPT-induced Cav1.2 reduction (n = 6). **P＜0.01, ANOVA.

Figure S3: The effect of synthesized PEST peptides on Cav1.2 expression. (A) Immunofluorescent images show that the individual PEST peptide bearing GFP is located in the soma and the axon hillock of primary cortical neurons. (B&C) Does-response (B, n = 4) and time-course (C, n = 3) of Cav1.2 protein in primary cortical neurons treated with synthesized PEST1 peptide. The concentration of PEST1 used for time-course was 150μM. (D&E) Does-response (D, n = 3) and time-course (E, n = 3) of Cav1.2 protein in primary cortical neurons treated with synthesized PEST3 peptide. The concentration of PEST3 used for time-course was 150μM. * P < 0.05, ** P < 0.01, compared with control, ANOVA.

Figure S4. Schematic diagram depicting the possible mechanisms through which ERα reduces Cav1.2 protein. Activation of ERα by PPT promotes Mdm2 association with Cav1.2. The subsequent Cav1.2 degradation by UPS involves K29-linked UB chains and the PEST motif. In APP/PS1 mice, the reduced Cav1.2 may contribute to ERα-induced cognitive improvement.
